# Effects of Partial Pulp Removal on Microbial Dynamics and Metabolite Profiles During On-Farm Cocoa Fermentation

**DOI:** 10.3390/foods15172976

**Published:** 2026-08-25

**Authors:** Gilson C. A. Chagas-Junior, Nelson Rosa Ferreira, Paulo Túlio de Souza Silveira, Anderson Moraes da Silva, Diego Assis das Graças, Artur Silva, Braian Saimon Frota da Silva, Dominic Wimmer, Priscilla Efraim, Alessandra Santos Lopes

**Affiliations:** 1 Graduate Program in Food Science and Technology (PPGCTA), Institute of Technology (ITEC), Federal University of Pará (UFPA), Campus Guamá, Belém 66075-110, PA, Brazil; nelson.ufpa@gmail.com (N.R.F.); braiansaimon@yahoo.com.br (B.S.F.d.S.); 2 Laboratory of Fruits and Vegetables, Department of Food Engineering and Technology, School of Food Engineering, Universidade Estadual de Campinas (Unicamp), Campinas 13083-862, SP, Brazil; ptssilveira@gmail.com (P.T.d.S.S.); pris@unicamp.br (P.E.); 3 Biological Engineering Laboratory (ENGEBIO), Innovation Space, Guamá Science and Technology Park, Belém 66075-110, PA, Brazil; dasilva.m.anderson@gmail.com (A.M.d.S.); diego.a87@gmail.com (D.A.d.G.); arturluizdasilva@gmail.com (A.S.); 4 Fraunhofer Institut für Verfahrenstechnik und Verpackung IVV, 85354 Freising, Germany; dominic.wimmer@ivv.fraunhofer.de

**Keywords:** *Saccharomyces cerevisiae*, chocolate, *Theobroma cacao*, fermentation, bacteria, yeasts

## Abstract

Partial cocoa pulp removal may modify substrate availability and affect microbial and metabolic dynamics during fermentation. This study evaluated the effects of beans with pulp and depulped beans on microbial dynamics and metabolite profiles during on-farm cocoa fermentation. Three treatments were evaluated for 144 h: CT, 100% beans with pulp; TA, 75% beans with pulp and 25% depulped beans; and TB, 50% beans with pulp and 50% depulped beans. Physicochemical parameters, culturable microbial populations, selected microbial isolates, sugars, and organic acids were analyzed throughout fermentation. Partial pulp removal was associated with differences in sugar and organic acid profiles, while temperature, pH, and titratable acidity showed the characteristic temporal progression of cocoa fermentation, with significant differences among treatments and fermentation times. *Saccharomyces cerevisiae* was frequently identified among selected yeast isolates, whereas *Acetobacter* sp. and *Fructobacillus pseudoficulneus* were among the bacterial taxa identified. Exploratory principal component and hierarchical cluster analyses indicated that fermentation time was the main factor associated with sample differentiation, while partial pulp removal showed secondary associations with physicochemical and microbiological profiles. Overall, partial pulp removal was associated with differences in microbial and metabolic profiles, indicating its potential for further investigation as a strategy to modulate cocoa fermentation.

## 1. Introduction

*Theobroma cacao* L. is a perennial species that produces cocoa fruits, with the main harvest in Pará state, Brazil, typically occurring in October and a smaller harvest in March [1]. Motamayor et al. [2] demonstrated that the species originates from the Amazon region and proposed its classification into ten genetic groups: *Amelonado*, *Contamana*, *Criollo*, *Curaray*, *Guiana*, *Iquitos*, *Marañon*, *Nacional*, *Nanay* and *Purús*. Cocoa processing begins with fruit harvesting, manual seed removal, and the microbial fermentation of the pulp and seeds [3].

Brazil is among the world’s leading cocoa producers, and cultivation of this crop holds considerable economic and social importance across several tropical regions. Despite its significance, the production chain traditionally focuses almost exclusively on the seeds for chocolate manufacturing, while the pulp surrounding the seeds is used primarily to a limited extent during fermentation [4]. The pulp, when removed before cocoa seed fermentation, is generated as a co-product for the production of frozen pulp, jams and beverages. To manufacture these products, additional processing steps are required, which are common for fruit pulps in general. In response to the increasing demand for functional, sustainable, and innovative foods, cocoa pulp has gained attention as a nutrient-rich yet underutilized source of bioactive compounds. Cocoa pulp consists predominantly of water (~80%), soluble carbohydrates (glucose, fructose, and sucrose), organic acids (notably citric and malic acids), and soluble fiber [5]. It also provides appreciable amounts of vitamins, particularly vitamin C and B-complex vitamins, as well as essential minerals such as potassium, magnesium, and calcium [6].

Partial pulp removal may alter the fermentation environment by modifying substrate availability and, consequently, influence temporal changes in culturable microbial groups and the occurrence of selected taxa during cocoa fermentation. Such effects may be reflected in changes in culturable microbial groups and in the occurrence of selected taxa during fermentation, as also suggested by a recent study in Bahia state (Brazil), where chocolates produced from partially depulped beans showed improved flavor and aroma in sensory analysis compared to those from non-depulped beans [7].

Microbial diversity during cocoa fermentation is influenced by the specific locality and prevailing process parameters (e.g., nutrient availability, pH, temperature, and oxygen levels), which directly affect the metabolic activity of the microbial population, including yeasts and bacteria. Numerous studies have documented a broad range of yeast and bacterial species involved in cocoa fermentations across different countries [8,9].

Recent studies have shown that *Saccharomyces cerevisiae*, species of the genus *Pichia*, and lactic acid bacteria belonging to the genera *Pediococcus*, *Lentilactobacillus*, and *Lacticaseibacillus* are among the microorganisms frequently reported in cocoa fermentation in the Brazilian Amazon [10,11].

Yeasts play a central role during cocoa fermentation because their metabolism contributes to sugar consumption and ethanol production, providing an important substrate for the subsequent metabolism of acetic acid bacteria (AAB) [3,8]. Their metabolic activities also contribute to the formation of volatile compounds, including aldehydes, esters, and ketones, which are associated with fruity and floral aroma notes and contribute to the development of the characteristic sensory properties of cocoa [3].

LAB may also contribute to the development of the fermentation environment through their metabolic activities. Moreover, recent research on tucupi fermentation, a traditional fermented product from the Brazilian Amazon, highlighted the role of *Pediococcus* in the fermentation process and its association with changes in undesirable biogenic amines, including putrescine and cadaverine, as well as in the potentially pathogenic microbiota [12]. Although these effects were reported for *tucupi* fermentation and cannot be directly extrapolated to cocoa fermentation, they illustrate the potential functional relevance of LAB during the fermentation of Amazonian food matrices.

During the intermediate and final stages of cocoa fermentation, acetic acid bacteria (AAB) generally become more prevalent as oxygen becomes available through periodic mixing of the fermenting mass. Under aerobic conditions, these bacteria oxidize the ethanol previously produced by yeasts into acetic acid, an exothermic process that contributes to the progressive increase in fermentation temperature. Acetic acid subsequently diffuses into the cocoa cotyledons, where, together with elevated temperature and other metabolites, it promotes embryo death and initiates biochemical reactions responsible for the formation of chocolate flavor precursors [3,5].

Although microbial dynamics during cocoa fermentation have been extensively investigated, previous studies have mainly focused on describing microbial succession, evaluating starter cultures, or improving fermentation quality under conventional processing conditions [13,14,15]. Comparatively less attention has been devoted to understanding how different levels of pulp removal modify the fermentation environment by altering substrate availability and, consequently, microbial dynamics and metabolite production under on-farm fermentation conditions. Because cocoa pulp constitutes the primary carbon source supporting microbial metabolism, modifying the amount of pulp retained around the beans may influence the physicochemical evolution of fermentation and the activity of culturable microbial groups [5]. However, these relationships remain insufficiently understood under practical cocoa production conditions.

In addition to its potential effects on fermentation, partial pulp removal may provide an opportunity for the recovery of cocoa pulp as a potentially valorisable coproduct. Previous studies have demonstrated the feasibility of using cocoa pulp in value-added food and beverage applications [16,17]. However, the technological, economic, and environmental feasibility of pulp valorization depends on subsequent processing and was not evaluated in the present study. Such potential applications may be considered within the broader context of circular bioeconomy approaches and the United Nations Sustainable Development Goals (SDGs), particularly SDG 12 (*Responsible Consumption and Production*).

Therefore, this study aimed to evaluate the effects of partial pulp removal on temporal changes in culturable microbial groups and the occurrence of selected microbial taxa, as well as on sugar dynamics, organic acid profiles, and physicochemical changes during cocoa fermentation in the Brazilian Amazon.

## 2. Materials and Methods

### 2.1. Fermentation Assay

The experiment was conducted during a single on-farm cocoa fermentation cycle at a commercial farm in the Brazilian Amazon under the production conditions routinely adopted by the producer.

**Figure 1:**
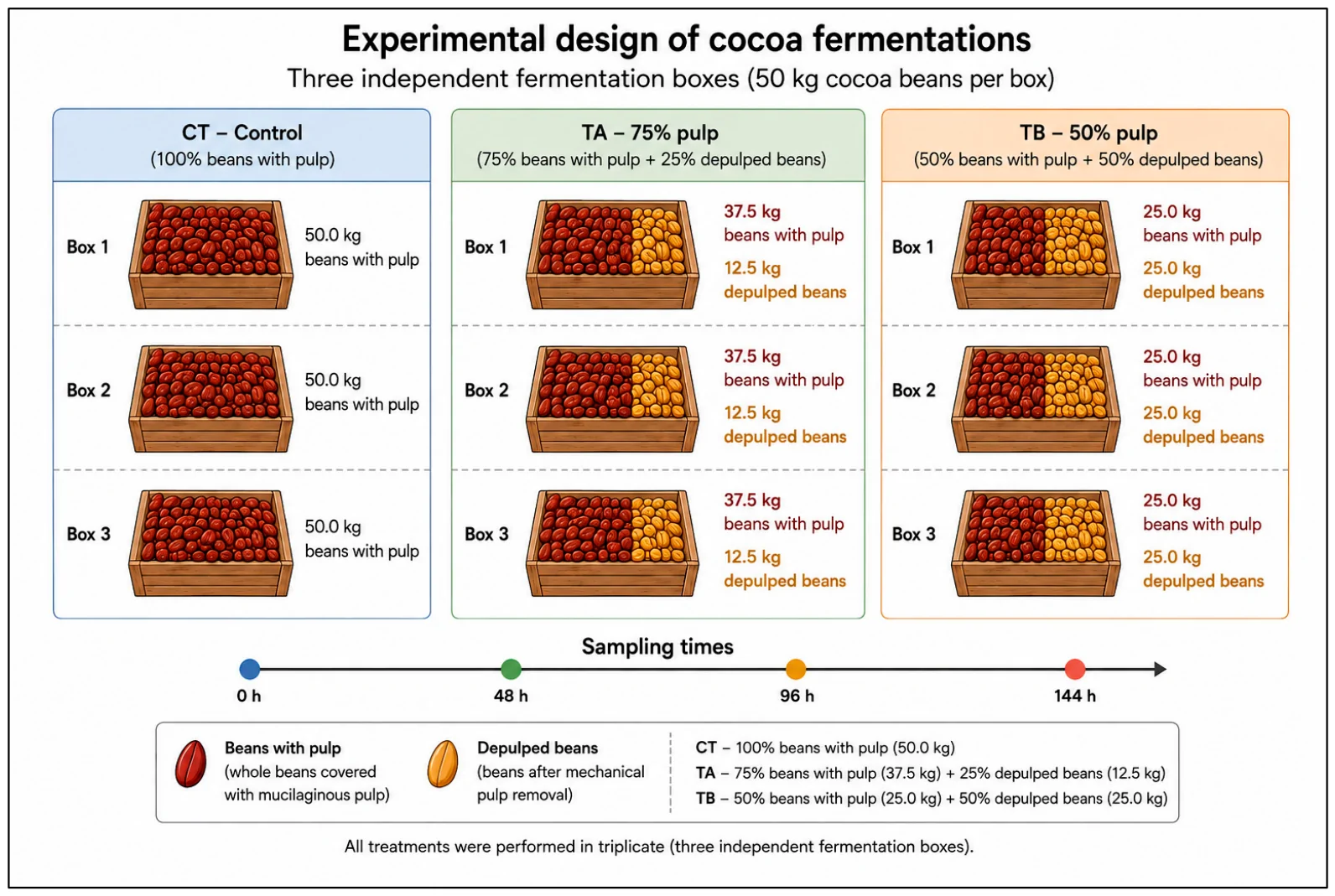
Experimental design of cocoa fermentations.

The cocoa fruits (*Forastero* group) were manually opened with stainless steel knives, and the seeds along with their pulp were collected. Spontaneous fermentation was carried out in triplicate, for each treatment, at Dois Irmãos Farm, in Moju City, Pará, Brazil (latitude 02°02′07.554″ S and longitude 48°42′28.536″ W). Following pulp removal using a Metvisa fruit pulper (model DG.10, Brusque, SC, Brazil), the seeds were distributed into wooden fermentation boxes for three different treatments: CT—100% beans with pulp; TA—75% beans with pulp + 25% depulped beans; TB—50% beans with pulp + 50% depulped beans (Figure 1). No visible damage to the husk or cotyledons was observed during processing.

Approximately 22 kg of cocoa pulp were recovered during the depulping procedure and immediately stored under frozen conditions for potential future studies. The recovered pulp was not further processed, characterized, or used for product development within the scope of the present study.

Each fermentation box received approximately 50 kg of cocoa beans and was covered with banana leaves. All fermentation boxes were subjected to the same turning schedule to ensure consistent aeration throughout the experiment period (144 h, or six days). The first turning was performed after 48 h of fermentation and subsequent turnings were carried out every 24 h using the same handling procedures and intensity for all treatments. Thus, turning was maintained as a standardized operational condition, allowing the effects of different pulp removal levels to be evaluated independently of aeration management. All procedures followed the traditional fermentation practices routinely employed by the producer, without intervention by the researchers.

The internal temperature of the fermenting cocoa mass was monitored throughout the fermentation process using a digital thermohygrometer (Instrutherm HT-600, São Paulo, Brazil). Temperature measurements were performed at each sampling time in the three independent fermentation boxes of each treatment. Within each fermentation box, three measurements were obtained at different locations of the fermenting mass to minimize spatial variability.

The cocoa fruits used in this study were registered in the Brazilian National System for the Management of Genetic Heritage and Associated Traditional Knowledge (SisGen; registration no. AC104A5).

### 2.2. Sampling, Physicochemical Analyses and Microbiological

Fermentation was performed in triplicate using three independent fermentation boxes per treatment. At each sampling time, samples were collected independently from each fermentation box. Samples used for physicochemical analyses were processed separately for each fermentation box.

Approximately 400 g of fermenting cocoa mass were manually collected from each treatment at 48 h intervals [11]. Of this quantity, 200 g was stored at −18 ± 2.0 °C for physicochemical analyses, and 200 g was stored at 8.0 ± 2.0 °C for microbiological assays. For pH determination (AOAC method 970.21) and total titratable acidity (TTA; AOAC method 31.06.06), the peel and germs of cocoa beans were removed, and the cotyledons were ground using an analytical mill (A11, IKA, Staufen, Germany). All analyses were performed in triplicate [18].

For microbiological analyses, equal aliquots collected from the three independent fermentation boxes of each treatment and sampling time were pooled to obtain a representative composite sample prior to serial dilution, plate counting and microbial isolation. Therefore, microbiological counts represent descriptive values of the pooled fermentation mass and were not used to estimate between-box variability [9].

Around 20 g of each refrigerated sample was homogenized in 180 mL of 1% saline peptone water (pH 7.2, Kasvi, Pinhais, PR, Brazil) for 2 min to obtain a 10^−1^ dilution. Serial decimal dilutions were subsequently prepared up to 10^−8^.

Aliquots of 0.1 mL from each dilution were plated in duplicate using the spread plate technique. For yeast enumeration, samples were inoculated on YPD agar (20 g/L glucose, 20 g/L bacteriological peptone, 10 g/L yeast extract, 15 g/L bacteriological agar, 100 mg/L chloramphenicol; pH 5.8); lactic acid bacteria (LAB) were isolated on MRS agar (Kasvi, pH 6.5) supplemented with 0.2% nystatin (Prati-Donaduzzi, Toledo, PR, Brazil) to inhibit fungal growth [19]. Acetic acid bacteria (AAB) were enumerated on GYEC agar (5% glucose, 1% yeast extract, 3% ethanol, 1.5% CaCO_3_, 1.5% bacteriological agar; pH 6.0), supplemented with 0.2% nystatin [20]. Plates were incubated at 30 °C for 72 h and 120 h (yeasts and AAB, respectively) and at 35 °C for 48 h (LAB). The microbial counts were expressed as log_10_ CFU/g of cocoa seeds of each microorganism. For each fermentation time point, colonies were obtained per plate, from which ten were randomly selected. The isolates were twice purified by streaking on sterile YPD agar (yeasts) and GYEC agar (AAB) plates and incubated at 30 °C for 72 h, and on MRS agar plates for LAB at 35 °C for 48 h.

Following the culture-dependent enumeration of LAB and AAB, colonies from countable plates were considered for bacterial isolation. Approximately 30 colonies were present on each countable plate, from which 10 colonies were randomly selected for preliminary biochemical characterization, corresponding to approximately 35% of the colonies recovered. This sampling approach was based on the procedure described by Papalexandratou et al. [21].

Selected colonies were purified and subjected to Gram staining and catalase testing. Isolates showing a Gram-positive and catalase-negative profile were considered presumptive LAB, whereas isolates showing a Gram-negative and catalase-positive profile were considered presumptive AAB [22]. Colonies that did not meet the corresponding phenotypic criteria were not considered for identification within the respective bacterial group.

Following the preliminary phenotypic characterization, a subset of the presumptive LAB and AAB isolates was selected for molecular identification. Genomic DNA was extracted from the selected isolates and the bacterial 16S rRNA gene was amplified by PCR using the primers described in the next section.

The molecularly identified isolates represent a selected subset of the culturable bacterial isolates and should therefore not be interpreted as representing the relative abundance of individual taxa in the total culturable bacterial population.

### 2.3. DNA Extraction, PCR, Taxonomic Classification, and Phylogenetic Analysis

DNA extraction of the isolated colonies was performed using the phenol/chloroform/isoamyl alcohol method [23], and the DNA was quantified using a NanoDrop spectrophotometer (Thermo Fisher Scientific, Waltham, MA, USA). The 16S rRNA gene was amplified using the forward primer 8F (5′-AGAGTTTGATCATGGCTCAG-3′) and reverse primer 1492R (5′-GGTTACCTTGTTACGACTT-3′). The ITS1-5.8S-ITS2 regions of rDNA were amplified using the primers ITS1 (5′TCCGTAGGTGAACCTGCGG3′) and ITS4 (5′TCCTCCGCTTATTGATATGC3′) [24,25,26]. The Polymerase Chain Reaction (PCR) was performed in a final volume of 25 µL, containing 0.5 to 10 ng of template DNA, 1 µM of each primer, dNTPs 0.2 mM, MgCl_2_ 2.5 mM, and 2.5 U of Taq DNA polymerase (Invitrogen, Waltham, MA, USA). Cycling was performed in the GeneAmp 9700 (Thermo Fisher Scientific) with an initial step of denaturation at 94 °C for 5 min, followed by 30 cycles of 94 °C for 1 min, 55 °C for 1 min, and 72 °C for 1 min, and a final step of 72 °C for 10 min [24,25,26]. The amplicons were sequenced on the ABI 3500 platform (Thermo Fisher Scientific). All PCR amplifications performed for the isolates selected for molecular identification were successful. The amplified products were subsequently subjected to sequencing, and the resulting sequences were compared with reference sequences using BLAST (Basic Local Alignment Search Tool, https://blast.ncbi.nlm.nih.gov/Blast.cgi, accessed on 21 August 2026). Taxonomic assignments were based on sequence similarity and phylogenetic analysis, as described below.

The sequences were compared to the 16S rRNA and ITS databases from GenBank using BLASTn. The 16S rRNA and ITS1-5.8S-ITS2 sequences were aligned using ClustalW software v. 2.1. Phylogenetic inferences were based on the Maximum Parsimony (MP) method. Initial trees were obtained by the random addition of sequences (10 replicates), and heuristic search was conducted using the Subtree-Pruning-Regrafting (SPR) algorithm with level 1 [27]. Branch robustness was evaluated through bootstrap analysis with 1000 replicates [28]. Branches with bootstrap support below 50% were collapsed. The consensus tree was visualized and edited using MEGA11 and FigTree v1.4.4 [29].

### 2.4. High-Performance Liquid Chromatography (HPLC) Analysis for Organic Acids and Sugars

Approximately 10 g of whole cocoa seeds (approximately 4–5 seeds, depending on seed size) from each treatment were placed in 50 mL conical tubes containing 10 mL of sterile ultrapure water and homogenized using a vortex mixer for 2 min, following previously reported procedures for cocoa fermentation samples. The tubes were centrifuged (8000× *g*, 4 °C, 10 min.), and the supernatant was transferred to a new tube. The extraction procedure was repeated once under the same conditions, totaling approximately 20 mL of extract [13,30,31].

Subsequently, aliquots of the extract (2 mL) were filtered through 0.22 µm PTFE hydrophilic syringe filters (Filtrilo, Colombo, PR, Brazil) into microtubes and stored at −18 °C until analysis.

Organic acids (citric, lactic, and acetic acids) and sugars (glucose, fructose, and sucrose) were quantified by High-Performance Liquid Chromatography (HPLC), using a Thermo Fisher Scientific Accela system equipped with an Aminex HPX-87H column (Bio-Rad Laboratories, Hercules, CA, USA) and a corresponding guard column. The column temperature was maintained at 45 °C and the mobile phase consisted of a 5 mM H_2_SO_4_ solution (pH 2.6) delivered at a flow rate of 0.6 mL/min. Detection was performed using a refractive index (RI) detector, and the total chromatographic run time was 25 min [32].

Metabolites were identified based on retention times obtained from authentic standards purchased from Sigma-Aldrich Chemical Co. (St. Louis, MO, USA): lactic acid, citric acid, and acetic acid; glucose, fructose, and sucrose. Quantification was performed using external calibration curves (Table 1), and the results were expressed as mg/g, wet basis (w.b.), of sample. All analyses were carried out in triplicate.

### 2.5. Statistical Analysis

All experiments were conducted using three independent fermentation boxes per treatment (*n* = 3). Data are presented as mean ± standard deviation (SD). For each response variable, the effects of treatment (CT, TA, and TB), fermentation time (0, 48, 96, and 144 h), and their interaction were evaluated using a linear mixed-effects model. Treatment, fermentation time, and the treatment × fermentation time interaction were included as fixed effects, whereas fermentation box was included as a random effect to account for repeated observations obtained from the same fermentation box over time. Pairwise comparisons among treatments within each fermentation time were performed with Holm–Bonferroni adjustment for multiple comparisons. Differences were considered statistically significant at *p* < 0.05. Statistical analyses were performed using Python 3.13.5 with the statsmodels package (version 0.14.6).

For microbiological analyses, samples collected from the three fermentation boxes belonging to the same treatment and sampling time were pooled prior to microbial enumeration and isolate identification. Therefore, microbiological results were interpreted descriptively and were not used to estimate biological variability among fermentation boxes.

Multivariate analyses were performed in a Google Colaboratory environment (Google Colab) using the Python programming language. Physicochemical and microbiological data were standardized by z-score transformation, considering samples as observations and analytical variables as descriptors. Principal Component Analysis (PCA) was applied to reduce data dimensionality and identify the variables associated with sample discrimination based on score and loading plots. Hierarchical Cluster Analysis (HCA) was performed using Euclidean distance and Ward’s linkage method to evaluate similarities among samples from the CT, TA and TB treatments.

PCA and HCA were performed using the mean physicochemical profiles obtained for each treatment at each fermentation time. Therefore, these multivariate analyses were used as exploratory tools to visualize patterns of similarity and variable association among samples and were not intended for inferential statistical testing.

## 3. Results and Discussion

### 3.1. Physicochemical Parameters

Total titratable acidity (TTA) increased throughout fermentation in all treatments, although the magnitude of these changes differed among treatments and fermentation times (Table 2). Differences among CT, TA, and TB were time-dependent, consistent with the significant treatment × fermentation time interaction observed in the statistical analysis. These patterns may reflect differences in substrate availability associated with the proportion of beans with pulp and depulped beans, together with the metabolic changes occurring throughout fermentation.

The pH and TTA are important indicators of fermentation performance and are commonly used to assess cocoa bean quality. According to the Cocoa Index proposed by Araujo et al. [33], the cocoa beans from all treatments did not meet the recommended pH and acidity values (5.60–6.57, and 10.53–19.37 meq. NaOH/100 g, respectively). However, this comparison should be interpreted with caution, since the present study evaluated fresh fermented beans immediately after fermentation, whereas the Cocoa Index was established using fermented and dried cocoa beans.

Drying is a critical post-fermentation step that significantly modifies the chemical composition of cocoa beans. During this process, volatile compounds, particularly organic acids such as acetic acid, may be partially removed through evaporation, resulting in increased pH values and reduced TTA. Therefore, the relatively low pH values and high TTA observed in our study may reflect the absence of the drying stage rather than inadequate fermentation. A similar observation has been reported for cocoa fermentations conducted in the Brazilian Amazon [1,19].

Furthermore, pH values above 5.0 favor the activity of endogenous proteases responsible for generating peptides and free amino acids, which are important precursors of chocolate flavor and aroma [34]. Considering that pH typically increases during drying as a consequence of acid loss, it is possible that the cocoa beans evaluated in our study may reach conditions more favorable for proteolytic activity during post-fermentation processing. This phenomenon is also associated with the formation of flavor precursors that participate in Maillard reactions during the roasting step, contributing to the development of desirable chocolate sensory attributes [3,5].

The fermentation temperature profiles (Table 2) support the interpretation of TTA and pH changes observed throughout the process. In all treatments, the progressive increase in temperature indicates active microbial metabolism, which is consistent with the physicochemical changes occurring during cocoa fermentation, including the increase in TTA and reduction in pH [3,13,19].

Although all fermentation masses reached temperatures typical of cocoa fermentation, differences in TTA values among treatments were consistent with differences in fermentation intensity associated with substrate availability rather than impairing the overall fermentation process. These findings indicate that temperature reflected the occurrence of active fermentation, whereas TTA was more sensitive to the metabolic differences promoted by the distinct levels of pulp reduction.

### 3.2. Identification of Yeasts and Bacteria During Cocoa Fermentation

**Figure 2:**
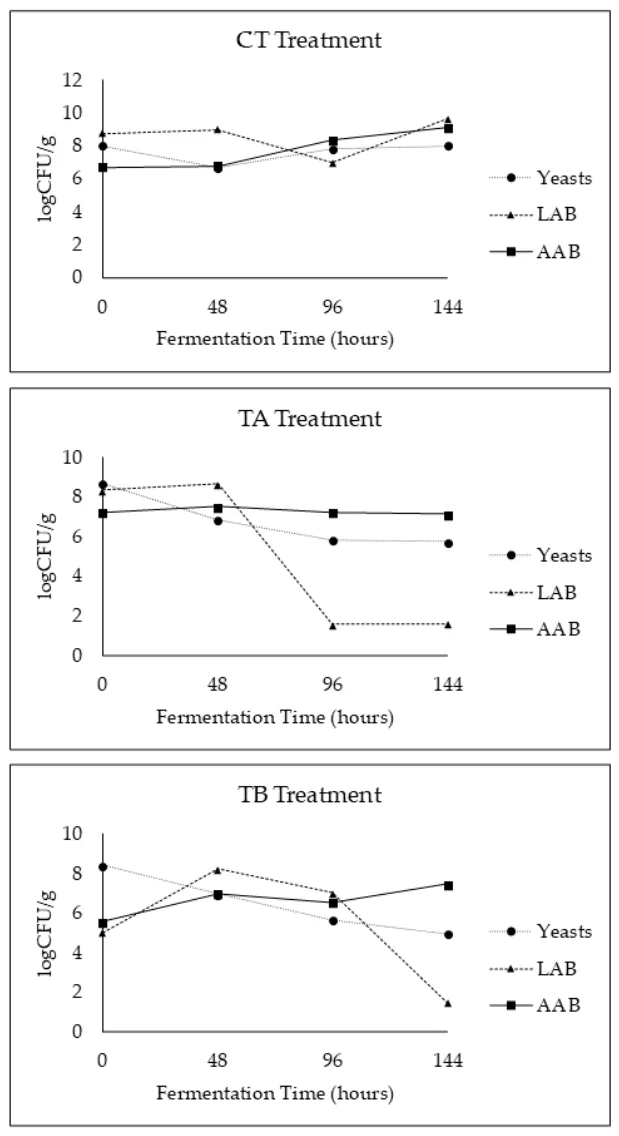
Dynamics of yeasts, LAB and AAB during cocoa fermentation treatments: CT—100% beans with pulp; TA—75% beans with pulp + 25% depulped beans; TB—50% beans with pulp + 50% depulped beans. Microbial counts were determined from pooled samples representing the three fermentation boxes for each treatment and sampling time. Therefore, the results are presented as descriptive profiles of microbial population dynamics.

The dynamics of the culturable microbial populations across the three fermentation treatments are presented in Figure 2.

In the control treatment (CT), the yeasts displayed patterns consistent with previous studies in the Amazon region [10,11], with yeast growth initially slowing and resuming around days 5–6. This resurgence likely reflects the composition of the surrounding pulp, including available fermentable sugars (glucose and sucrose) and organic acids produced by lactic and acetic acid bacteria, which can hydrolyze sucrose into glucose and fructose, thereby providing additional substrate for yeast proliferation in the later stages of fermentation [19].

In contrast, yeast populations in the TA and TB treatments were markedly lower, likely due to the reduced pulp content of these seeds.

In our study, *Saccharomyces cerevisiae* was the most frequently isolated species in all treatments, followed by *Hanseniaspora opuntiae* and *Pichia kudriavzevii* during the cocoa fermentation (Table S1 in Supplementary Material, Table 3 in main text). These species are notable for producing a wide range of esters, alcohols, and aldehydes that contribute desirable ‘fruity’, ‘floral’, and ‘sweet’ aromas to chocolate [15,35]. These species are highly adaptable to diverse environmental conditions and can suppress the growth of spoilage microorganisms [36]. Chagas Junior et al. [19] reported that the use of starter cultures combining *S. cerevisiae* and *P. kudriavzevii* during cocoa fermentation was associated with changes in undesirable compounds, formation of volatile compounds relevant to cocoa aroma, and improved fermentation performance, including a reduction in fermentation time. These findings suggest that the interaction between these two yeast species may have beneficial effects under the fermentation conditions investigated; however, such effects should not be generalized to all yeast taxa recovered during cocoa fermentation.

**Figure 3:**
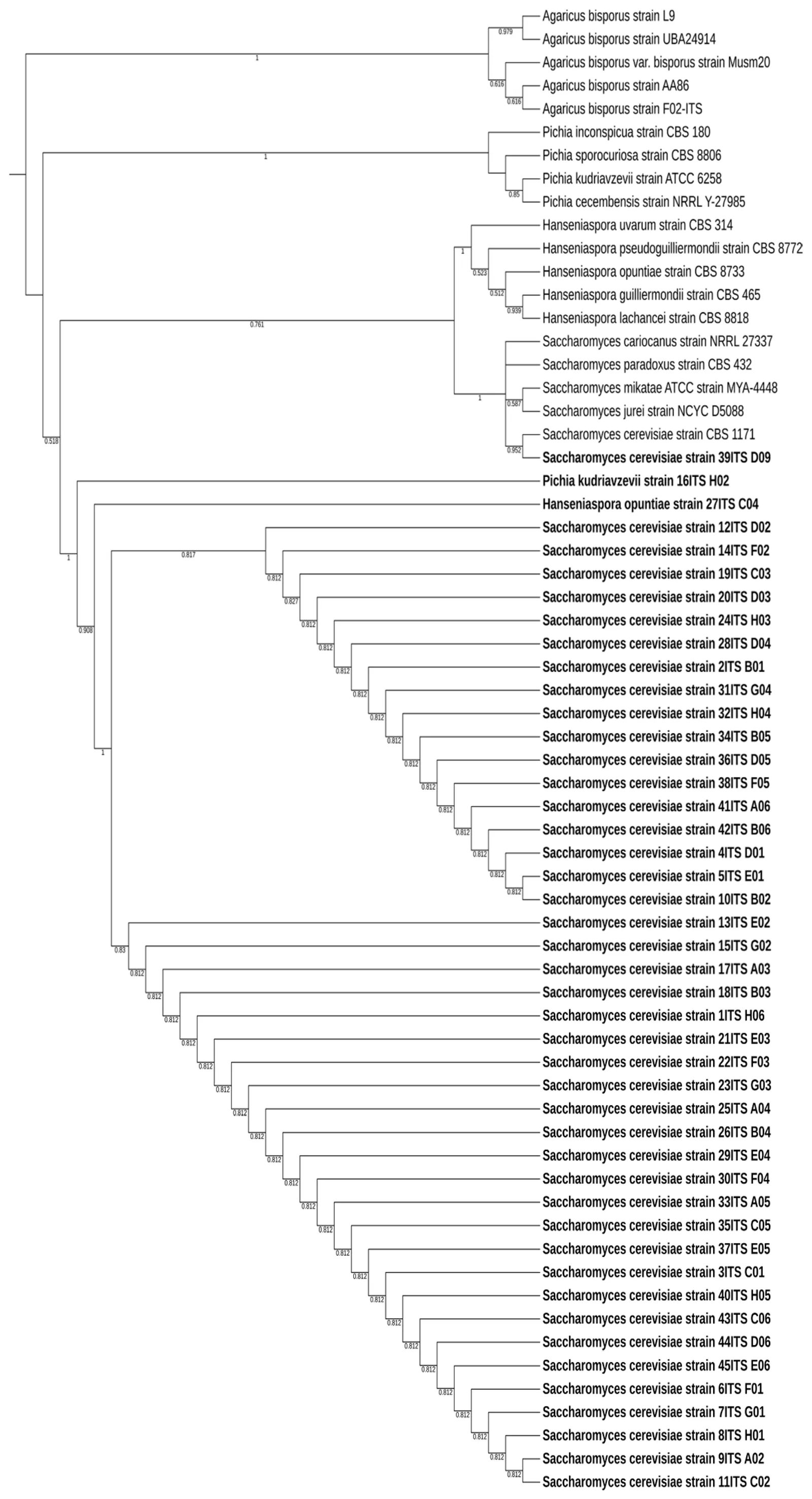
Phylogenetic tree based on the ITS region of all yeasts identified in this study. The tree was inferred using the Maximum Parsimony (MP) method, with Subtree-Pruning-Regrafting (SPR) used as the heuristic search algorithm, and 1000 bootstrap replicates. Sequenced isolates are shown in bold, and the scale bar indicates the number of substitutions per site.

The general phylogenetic tree (Figure 3), constructed from ITS region sequences, provided an overview of the phylogenetic relationships among the yeasts identified in this study. The tree topology clearly confirmed the separation of the genera *Saccharomyces*, *Pichia*, and *Hanseniaspora*, with high bootstrap support at the major nodes. The majority of the selected isolates clustered with *Saccharomyces cerevisiae*, forming a well-supported and cohesive clade, indicating that *S. cerevisiae* was frequently recovered among the isolates selected for molecular identification. Isolates of *Pichia kudriavzevii* and *Hanseniaspora opuntiae* were positioned on distinct branches, reflecting their separate taxonomic identities. A similar frequent recovery of *S. cerevisiae* among selected isolates was reported during spontaneous cocoa fermentation in another city of Pará State, Tomé-Açu [11].

The ITS-based phylogenetic tree (Figure S1, Supplementary Material) revealed the presence of species from the genera *Hanseniaspora* (e.g., *H. opuntiae*, *H. guilliermondii*, *H. uvarum*) and *Wickerhamomyces anomalus.* Notably, *H. opuntiae* (isolate 27ITS C04) clustered with high bootstrap support (~0.70) alongside the reference strain CBS 8733, confirming the robustness of this identification. These non-*Saccharomyces* yeasts are commonly reported during the first stages of cocoa fermentation and are gradually replaced by *S. cerevisiae* as the process progresses [8,11].

The phylogenetic tree corresponding to *Pichia* (Figure S2, Supplementary Material) revealed the presence of *P. kudriavzevii* (isolate 16ITS H02), a species known for its significant fermentative activity and tolerance to adverse conditions. The clustering of this isolate with *P. kudriavzevii* ATCC 6258 and other reference strains confirmed its taxonomic identity. Notably, *P. kudriavzevii* is recognized for its ability to produce volatile compounds that contribute to the characteristic aroma of fermented cocoa and chocolate [14,35]. The *S. cerevisiae* phylogenetic tree (Figure S3, Supplementary Material) showed that the majority of sequenced isolates (e.g., 23ITS D04, 1ITS4_H06 to H10, etc.) clustered consistently with *S. cerevisiae*. The high sequence homology with reference strains confirmed the taxonomic assignment of these isolates to *S. cerevisiae*. Among the isolates selected for molecular identification, *S. cerevisiae* was the most frequently recovered taxon across the treatments.

Among the bacterial isolates selected for molecular identification, *Acetobacter* sp. (including *Acetobacter* sp. closely related to *A. oryzoeni*, *Acetobacter* sp. closely related to *A. tropicalis*, and *Acetobacter* sp. closely related to *A. oryzifermentans*) represented the most frequently recovered taxon, together with LAB such as *Fructobacillus pseudoficulneus* and *Lentilactobacillus farraginis* (Table S2, Supplementary Material). These taxa are consistent with bacterial groups commonly reported during cocoa fermentation, in which LAB are generally associated with early stages of fermentation and acetic acid bacteria with the subsequent oxidation of ethanol to acetic acid. Notably, the specific role of *F. pseudoficulneus* in cocoa fermentation remains poorly elucidated in the literature [37].

The detection of multiple acetic acid bacterial taxa indicates that several AAB taxa were recovered from the fermentation under the different pulp removal conditions, consistent with bacterial groups commonly reported during cocoa fermentation [3]. While treatments TA and TB showed reduced microbial counts, likely reflecting lower pulp availability, the persistence of culturable microbial populations, together with the observed pH and total titratable acidity (TTA) trends, indicates that fermentation-associated microbial activity was maintained under the altered substrate conditions. The identified AAB and LAB isolates are shown in Table S2 (Supplementary Material) and Table 4.

The increase in presumptive AAB counts observed after 48 h in all treatments is consistent with the turning operation, which promoted oxygen diffusion into the fermentation mass and favored the oxidative metabolism of these bacteria. Since all fermentation boxes were turned according to the same schedule procedure, aeration was considered equivalent among treatments. The pulp fraction in treatments TA and TB was sufficient to serve as a substrate for the remaining AAB population, which utilized the ethanol produced by the yeasts for their metabolism.

**Figure 4:**
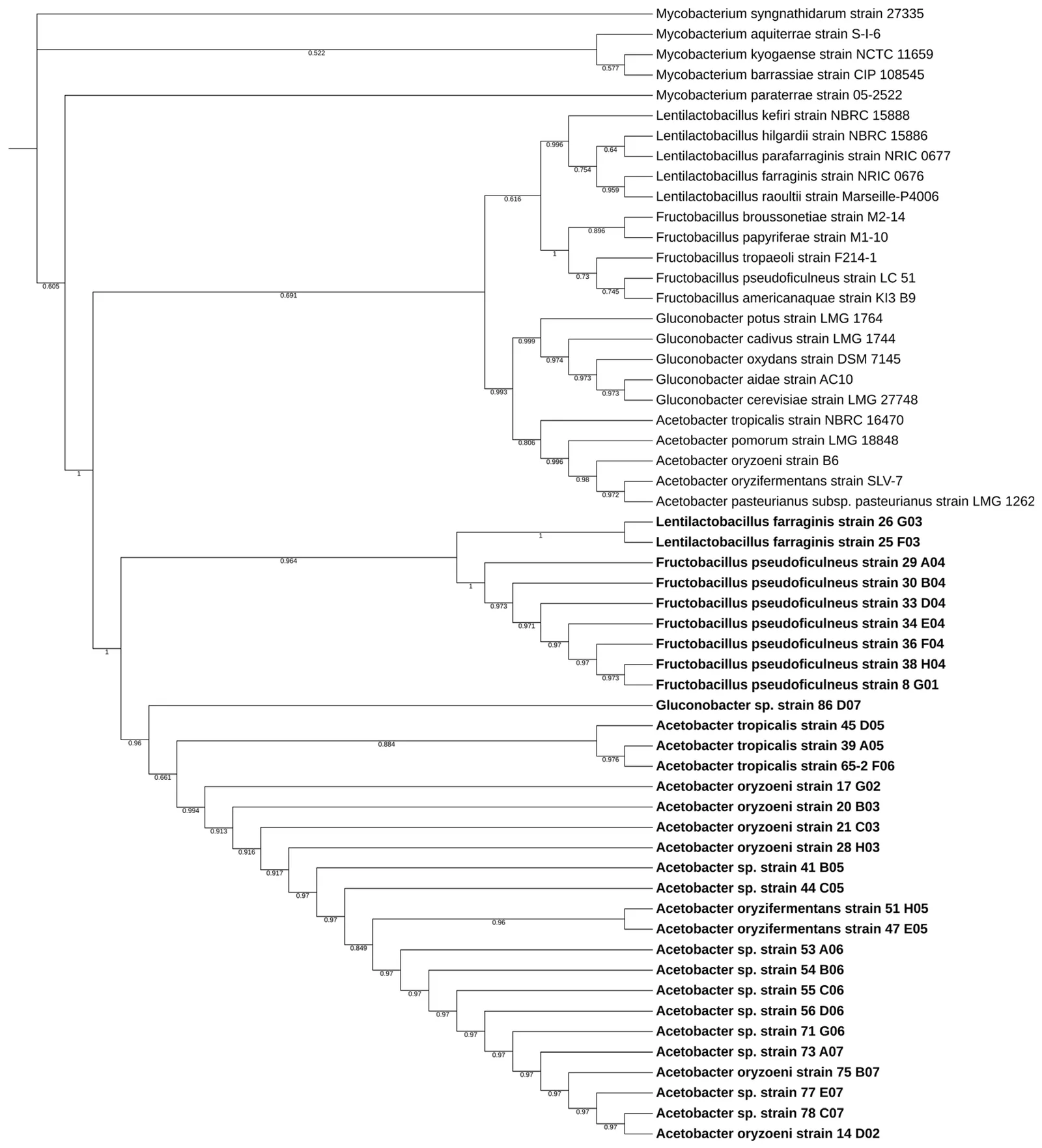
Phylogenetic tree based on the 16S region of all bacterial species identified in this study. The tree was inferred using the Maximum Parsimony (MP) method, with Subtree-Pruning-Regrafting (SPR) used as the heuristic search algorithm, and 1000 bootstrap replicates. Sequenced isolates are shown in bold, and the scale bar indicates the number of substitutions per site.

The general bacterial identification (Figure 4) encompassed a broad diversity of AAB and lactic acid bacteria. *Acetobacter* isolates (e.g., 14_D02, 20_B03, 28_H03) clustered with *Acetobacter* sp. closely related to *A. oryzoeni* and *A. oryzifermentans*, whereas *Acetobacter* sp. closely related to *A. tropicalis* (39_A05, 65-2_F06) formed a distinct clade with high bootstrap support (Figure S4, Supplementary Material). Regarding the lactic acid bacteria, *Fructobacillus pseudoficulneus* isolates (e.g., 8_G01, 29_A04, 38_H04) showed strong clustering with the type sequence NR_042753.1, confirming their identification (Figure S5, Supplementary Material). This genus is commonly found in fructose-rich environments, such as cocoa pulp, and additionally, isolates 25_F03 and 26_G03 were identified as *Lentilactobacillus farraginis*, clustering with sequence NR_041467.1.1. This species is associated with carbohydrate fermentation and may contribute to medium acidification [38].

It is important to acknowledge that ten isolates of *Acetobacter* were identified only at the genus level in this study. This limitation arises from the use of partial 16S rRNA gene sequencing, which, while effective for genus-level assignment, may not provide sufficient resolution for definitive species-level identification within the *Acetobacter* genus due to the high sequence similarity observed among closely related species [39].

Protein-coding housekeeping genes such as *dnaK*, *groEL*, and *rpoB* can provide greater resolution for species-level discrimination within the genus [39]. Despite this limitation, the genus-level identification does not compromise the primary conclusions regarding the occurrence of acetic acid bacteria during cocoa fermentation: *Acetobacter* spp. were recovered from all treatments and from multiple fermentation time points, supporting their occurrence under the different pulp removal conditions. These bacteria play an important role in cocoa fermentation by oxidizing yeast-derived ethanol to acetic acid, contributing to the metabolic and physicochemical changes characteristic of the process [3,5].

Furthermore, the recovery of multiple *Acetobacter* sp. isolates closely related to *A. oryzoeni*, *A. tropicalis*, and *A. oryzifermentans* indicates taxonomic diversity within this functional group. Therefore, while species-level identification would provide additional taxonomic resolution, the current data remain informative for understanding the occurrence of culturable bacterial taxa associated with cocoa fermentation under different pulp removal conditions.

Our findings contribute to the understanding of culturable microbial groups and selected bacterial taxa recovered during cocoa fermentation under different levels of pulp availability. The observed microbial counts, together with the recovery of bacterial isolates belonging to taxa commonly reported in cocoa fermentation, indicate that culturable microbial activity was maintained under the different pulp removal conditions. These findings suggest that partial pulp removal may modify the fermentation environment without preventing the recovery of microorganisms commonly associated with cocoa fermentation. However, because microbial identification was performed on a selected subset of isolates, these results should not be interpreted as evidence that partial pulp removal did not affect microbial succession or that the microbial community was resistant or resilient to changes in environmental conditions.

### 3.3. Organic Acids and Sugars Analyses by HPLC

The dynamics of organic acids during cocoa fermentation are presented in Table 5. Cocoa pulp naturally contains several organic acids, including succinic, malic, and citric acids [5]. Among these, citric acid was identified and quantified by HPLC throughout fermentation in all treatments. As expected, citric acid concentrations were highest at the beginning of fermentation (0 h), particularly in the control treatment (CT, 0.56 mg/g), followed by TA (0.47 mg/g) and TB (0.30 mg/g). The lower initial concentrations observed in TA and TB were directly associated with the partial removal of cocoa pulp prior to fermentation, since citric acid is predominantly present in the pulp fraction.

The progressive increase in citric acid observed in TA and TB treatments likely reflects changes in the balance between citrate availability and microbial utilization throughout fermentation rather than the abundance of a single microbial group. Partial pulp reduction modified the fermentation environment by decreasing the amount of mucilage surrounding the cocoa beans, thereby altering substrate availability and the overall metabolic activity of the fermenting microbiota [7]. Consequently, the higher residual citric acid concentrations observed in both treatments may result from a combination of reduced citrate consumption and changes in the physicochemical conditions governing citrate metabolism. Although LAB are recognized as important microorganisms involved in citrate metabolism during cocoa fermentation [3], our study did not evaluate citrate-metabolizing enzyme activity. Therefore, a direct relationship between LAB abundance and citric acid utilization cannot be established.

Several cocoa-associated LAB species possess citrate lyase activity and are capable of converting citric acid into lactic acid, acetic acid, mannitol and other metabolites that contribute to fermentation performance and flavor development [3,40,41]. Nevertheless, citrate metabolism depends not only on the presence of citrate-utilizing LAB, but also on microbial metabolic activity, substrate availability and environmental conditions throughout fermentation. Therefore, the citric acid profiles observed in our study should be interpreted as the result of multiple interacting processes influenced by partial pulp reduction rather than as the exclusive consequence of changes in LAB populations.

The changes in lactic and acetic acid concentrations were generally consistent with the microbial profiles observed during fermentation. Partial pulp removal in treatments TA and TB reduced the availability of fermentable substrates, which likely influenced the overall metabolic activity of the fermenting microbiota. In addition, the cocoa pulp removal procedure involved greater manual handling of the cocoa mass, which may have facilitated the natural inoculation of lactic acid bacteria (LAB) and acetic acid bacteria (AAB) from the surrounding environment to the fermenting beans [3,4]. This may explain the relatively high initial populations of these microbial groups observed in all treatments.

Although relatively low lactic acid concentrations were detected throughout fermentation, these results should be interpreted with caution. Organic acids were quantified from aqueous extracts obtained after homogenization of whole cocoa beans followed by centrifugation, and therefore represent only the fraction recovered by the extraction procedure rather than the total amount produced during fermentation. Because lactic acid may diffuse into the cotyledons or remain associated with the bean matrix, its recovery in the aqueous extracts may be incomplete [3,4]. Consequently, the low concentrations detected cannot be attributed solely to the relatively low abundance of LAB, but likely reflect both microbial metabolism and the limitations inherent to the extraction methodology employed in the present study.

In treatment TB (50% beans with pulp + 50% depulped beans), acetic acid concentrations increased during the later stages of fermentation, reaching the highest value at 144 h (3.30 mg/g). At this fermentation time, TB differed significantly from both CT and TA (*p* < 0.05). Although acetic acid is mainly produced through the oxidation of ethanol by acetic acid bacteria (AAB) during cocoa fermentation [3,4,5], the concentrations determined in the present study should be interpreted with caution.

Organic acids were quantified from aqueous extracts obtained after homogenization of whole cocoa beans and therefore represent the fraction recovered by the extraction procedure rather than the total amount produced during fermentation. In addition, the periodic turning of the fermentation mass after 48 h, and every 24 h thereafter, promoted aeration of the cocoa mass, creating conditions favorable for oxidative metabolism [3,8]. Consequently, the acetic acid concentrations measured in TB likely reflect the combined effects of microbial activity, aeration associated with the turning practice, diffusion of organic acids into the cotyledons, volatilization, and the extraction methodology employed, rather than a direct relationship with yeast abundance alone.

The sugars (Table 6) naturally present in cocoa pulp, particularly glucose and fructose, serve as important substrates for fermentative microorganisms, especially yeasts and LAB, during the initial stages of cocoa fermentation [3,4,5]. In our study, glucose concentrations progressively decreased throughout fermentation in the control treatment (CT), indicating its continuous utilization by the fermentative microbiota. In contrast, glucose levels remained relatively stable after 48 h in treatments TA and TB.

Partial pulp removal significantly reduced the initial concentrations of glucose and fructose in treatments TA and TB compared with CT, reflecting the lower availability of fermentable substrates resulting from cocoa pulp removal. Nevertheless, both sugar concentrations were reduced during fermentation in all treatments. The higher concentrations observed in CT throughout the process further demonstrate the contribution of cocoa pulp as the main source of readily fermentable carbohydrates. Since glucose and fructose are preferentially metabolized by yeasts and LAB, their reduction is consistent with their utilization by fermentative microorganisms and with the concurrent formation of ethanol, organic acids, and other fermentation metabolites [3].

In contrast, sucrose concentrations remained relatively stable throughout the 144 h fermentation period, varying only between 1.21 and 1.40 mg/g across all treatments. Instead, the major changes in sugar composition during fermentation were associated with glucose and fructose. Similar patterns have been reported in cocoa fermentation, where reducing sugars constitute the principal substrates supporting microbial growth and metabolic activity [3].

The present study focused exclusively on the fermentation stage of cocoa processing. Although partial pulp removal modified the fermentation environment and organic acid dynamics, the effects of these changes on flavor precursor development, volatile compound formation and the sensory quality of chocolate remain unknown. Since drying and roasting profoundly influence the chemical composition of cocoa beans, further studies integrating post-fermentation processing, volatile profiling and sensory evaluation are necessary to determine the technological implications of partial pulp removal on chocolate quality.

### 3.4. Multivariate Analysis (PCA and HCA)

**Figure 5:**
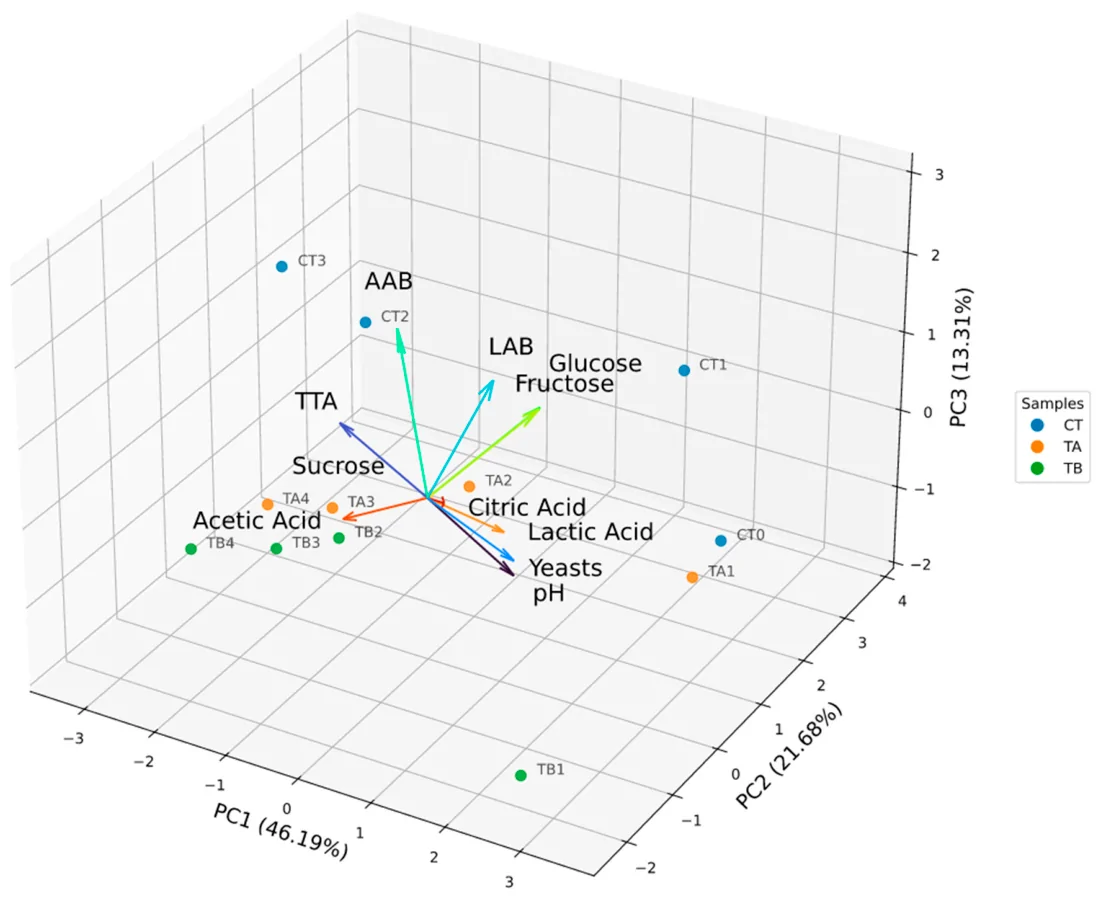
Principal Component Analysis of the physicochemical characteristics during the fermentation treatments: CT—100% beans with pulp; TA—75% beans with pulp + 25% depulped beans; TB—50% beans with pulp + 50% depulped beans. Notes: CT0, CT1, CT2, CT3: times 0, 48, 96 and 144 h of fermentation in the control treatment, respectively. TA1, TA2, TA3, TA4: times 0, 48, 96 and 144 h of fermentation in TA treatment, respectively. TB1, TB2, TB3, TB4: times 0, 48, 96 and 144 h of fermentation in TB treatment, respectively.

Principal Component Analysis (PCA) was performed to investigate the relationships among physicochemical, microbiological, sugar, and organic acid variables during cocoa fermentation under different pulp removal treatments. The first three principal components explained 81.18% of the total variance, with PC1, PC2, and PC3 accounting for 46.19%, 21.68%, and 13.31%, respectively (Figure 5).

The PCA indicated that sample differentiation was primarily associated with three major processes occurring during cocoa fermentation: acidification, substrate availability and utilization, and microbial dynamics. The first principal component (PC1), which explained 46.19% of the total variance, was positively associated with pH, yeast populations, LAB, and lactic acid, and negatively associated with TTA, acetic acid, sucrose, citric acid, and AAB. Therefore, PC1 mainly reflected the physicochemical changes associated with fermentation progression, particularly the inverse relationship between pH and acidity development. The opposite positioning of pH and TTA/acetic acid is consistent with the progressive acidification of the cocoa mass throughout fermentation [7,15,19].

The second PC (PC2), responsible for 21.68% of the total variance, was mainly influenced by fructose, glucose, citric acid, and sucrose. These variables represent the principal fermentable substrates available to the microbial community and indicate that PC2 was associated with differences in sugar availability and utilization among treatments and fermentation stages. The similar contributions of glucose and fructose suggest a correlated metabolic behavior of these sugars during fermentation, consistent with their simultaneous utilization by fermentative microorganisms.

PC3, which explained 13.31% of the variance, was predominantly associated with acetic acid bacteria (AAB) and lactic acid bacteria (LAB). Consequently, this component was associated with variation in culturable bacterial abundance and highlighted the contribution of microbial population to sample differentiation during fermentation. The strong contribution of these microbial groups suggests that variation in culturable bacterial populations contributed to sample differentiation during fermentation.

The score distributions revealed a clear temporal behavior. Samples collected during the initial stages of fermentation were generally associated with higher pH values and greater availability of sugars and citric acid [7], whereas samples from the later stages were associated with increased acidity and bacterial activity. This progressive displacement of samples along the PCs was consistent with temporal changes in the physicochemical and microbiological characteristics of the cocoa mass during fermentation.

Among the treatments, both partially depulped fermentations (TA and TB) exhibited progressive shifts in the multivariate space throughout fermentation, indicating continuous physicochemical and microbiological changes. The control treatment (CT) showed a distinct positioning during the final fermentation stages, particularly in relation to PC3, suggesting a stronger association with bacterial populations at advanced stages of fermentation.

Overall, the PCA showed that sample differentiation was primarily associated with acidification processes (PC1), substrate availability and utilization (PC2), and variation in culturable bacterial populations (PC3). These patterns were consistent with differences associated with fermentation time and pulp removal level in the physicochemical and microbiological characteristics of the fermentations. However, temporal progression was the main factor associated with sample differentiation.

**Figure 6:**
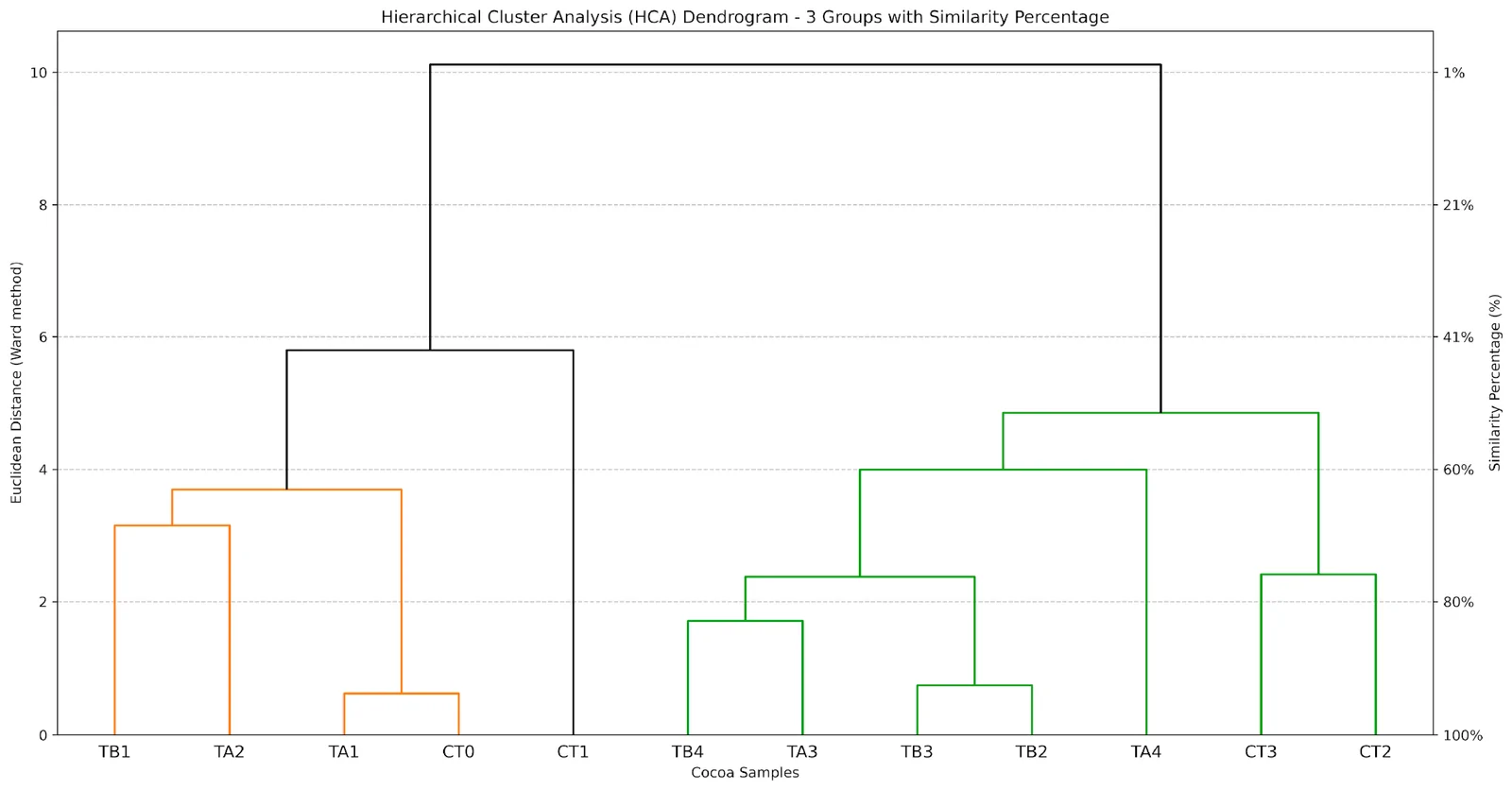
Hierarchical Cluster Analysis of the physicochemical characteristics during the fermentation treatments: CT—100% beans with pulp; TA—75% beans with pulp + 25% depulped beans; TB—50% beans with pulp + 50% depulped beans. Notes: CT0, CT1, CT2, CT3: times 0, 48, 96 and 144 h of fermentation in the control treatment, respectively. TA1, TA2, TA3, TA4: times 0, 48, 96 and 144 h of fermentation in TA treatment, respectively. TB1, TB2, TB3, TB4: times 0, 48, 96 and 144 h of fermentation in TB treatment, respectively.

Hierarchical Cluster Analysis (HCA, Figure 6) was performed to evaluate the multivariate similarity among cocoa samples subjected to different pulp removal levels throughout fermentation. The dendrogram revealed two major clusters corresponding predominantly to early/intermediate and advanced fermentation stages, indicating that fermentation time was the main factor associated with sample grouping.

The first cluster comprised mainly samples collected at the beginning of fermentation, including CT0, CT1, TA1, TA2, and TB1. The high similarity observed among some of these samples indicates that the physicochemical and microbiological profiles were relatively comparable during the early stages of fermentation, regardless of pulp removal level. The second cluster grouped samples collected at 96 and 144 h of fermentation, including CT2, CT3, TA3, TA4, TB2, TB3, and TB4. The formation of this cluster was consistent with the progressive changes observed during fermentation, including substrate depletion, organic acid accumulation, and changes in microbial populations. Within this group, some treatment-specific subclusters were observed, indicating that partial cocoa pulp removal was also associated with differences in fermentation profiles, although these differences were less pronounced than those associated with fermentation time.

Notably, CT2 and CT3 formed a distinct subcluster, suggesting that CT maintained particular physicochemical and microbiological characteristics during the advanced stages of fermentation. Likewise, similarities observed among samples from TA and TB were consistent with convergent fermentation profiles under certain fermentation conditions.

Overall, the HCA results were consistent with the patterns observed in the PCA, with fermentation progression being the main factor associated with sample differentiation. The clustering pattern was consistent with temporal changes in acidification, sugar concentrations, organic acid concentrations, and culturable bacterial populations during fermentation, whereas partial pulp removal showed a comparatively weaker association with the overall multivariate profiles.

The present study evaluated a single cocoa fermentation cycle conducted under the environmental and production conditions of one farm during the Amazonian summer. Therefore, the observed microbial dynamics and metabolite profiles should be interpreted within the context of the specific fermentation conditions investigated. Further studies involving different harvest seasons, production years, cocoa batches, and production systems are necessary to assess the reproducibility and broader applicability of these findings.

## 4. Conclusions

Partial pulp removal altered sugar availability, organic acid dynamics, and the abundance of culturable microbial groups during cocoa fermentation. Reduced substrate availability modified the fermentative environment, resulting in distinct microbial and metabolic profiles while preserving the overall progression of the fermentation process. Exploratory PCA and HCA indicated that fermentation time was the primary factor associated with sample differentiation, whereas partial pulp removal contributed to secondary variations in the physicochemical and microbiological profiles.

These findings suggest that partial pulp removal represents a promising technological strategy for cocoa fermentation. However, the present results are limited to the fermentation cycle evaluated under the conditions of this study, and further investigations involving different harvest seasons, production years, and production systems are required to evaluate the broader applicability of these findings. An additional limitation of the present study is that the microbial characterization was based exclusively on culture-dependent methods followed by the identification of selected isolates. Consequently, the results describe only the culturable fraction of the microbial community associated with cocoa fermentation and do not encompass non-culturable or low-abundance microorganisms that may also contribute to fermentation dynamics.

Future studies integrating sensory evaluation of chocolate made from these fermentations, culture-dependent approaches with culture-independent techniques, such as amplicon sequencing or metagenomic analyses, will provide a more comprehensive understanding of the temporal dynamics of the microbial community and help clarify how partial pulp removal influences the overall microbial ecology of cocoa fermentation.

## Figures and Tables

**Table 1:** Concentration ranges of the analytes used for construction of the calibration curves, together with the respective correlation coefficients and limits of quantification for HPLC analysis.

Compound	Concentration Range (g/L)	Linear Equation (R^2^)	LOD (mg/g)	LOQ (mg/g)
Acids				
Citric	0.1–10	y = 0.000000789917x + 0.276501 (>0.999)	0.01	0.03
Lactic	0.1–10	y = 0.000000803397x − 0.0281193 (>0.999)	0.01	0.02
Acetic	0.1–10	y = 0.00000116614x − 0.00917348 (>0.999)	0.02	0.05
Sugars				
Glucose	0.1–10	y = 0.000000491271x − 0.0273516 (>0.999)	0.03	0.08
Fructose	0.1–10	y = 0.000000531237x − 0.00936251 (>0.999)	0.03	0.08
Sucrose	0.1–10	y = 0.000000509505x − 0.0690702 (>0.999)	0.30	0.92

R^2^—Correlation coefficient of linear regression.

**Table 2:** Mean values * of fermentation temperature, pH and total titratable acidity (TTA) during the fermentation treatments: CT—100% beans with pulp; TA—75% beans with pulp + 25% depulped beans; TB—50% beans with pulp + 50% depulped beans.

Parameters/ Treatments	0 h	48 h	96 h	144 h
Temperature of Fermentation (°C)				
CT	30.5 ± 0.26 ^b^	38.2 ± 0.06 ^b^	46.8 ± 0.95 ^a^	44.8 ± 1.37 ^a^
TA	31.3 ± 0.85 ^ab^	37.7 ± 1.94 ^ab^	44.8 ± 2.11 ^a^	46.1 ± 0.91 ^a^
TB	31.9 ± 0.31 ^a^	42.8 ± 0.70 ^a^	45.5 ± 0.60 ^a^	43.7 ± 1.01 ^a^
pH				
CT	6.71 ± 0.03 ^b^	5.31 ± 0.03 ^a^	4.34 ± 0.01 ^b^	4.28 ± 0.02 ^b^
TA	6.87 ± 0.03 ^a^	5.31 ± 0.03 ^b^	4.53 ± 0.01 ^a^	4.25 ± 0.02 ^b^
TB	6.65 ± 0.04 ^c^	4.91 ± 0.02 ^b^	4.53 ± 0.02 ^a^	4.47 ± 0.03 ^a^
TTA ^1^				
CT	3.39 ± 0.08 ^ab^	13.14 ± 0.42 ^b^	35.45 ± 0.65 ^a^	40.00 ± 0.72 ^a^
TA	3.76 ± 0.35 ^a^	12.63 ± 0.37 ^b^	23.64 ± 0.72 ^c^	37.70 ± 3.21 ^b^
TB	1.39 ± 0.01 ^b^	18.31 ± 1.22 ^a^	29.86 ± 0.86 ^b^	35.76 ± 0.64 ^c^

* Values are expressed as mean ± standard deviation (*n* = 3 independent fermentation boxes). Different lowercase letters within the same fermentation time and variable indicate significant differences among treatments according to pairwise comparisons with Holm–Bonferroni adjustment (*p* < 0.05). ^1^ meq. NaOH/100 g: milliequivalent sodium hydroxide solution 0.1 N per 100 g sample.

**Table 3:** Values represent the number of selected isolates identified at each treatment and fermentation time and do not represent the relative abundance of each species in the total culturable yeast population.

Yeasts Species	Treatment	0 h	48 h	96 h	144 h	Total Isolates
*Saccharomyces cerevisiae*	CT	4	3	2	1	10
	TA	6	3	3	3	15
	TB	6	6	3	2	17
					Total	42
*Pichia kudriavzevii*	CT	1	0	0	0	1
	TA	0	0	0	0	0
	TB	0	0	0	0	0
					Total	1
*Hanseniaspora opuntiae*	CT	0	0	0	0	0
	TA	0	1	0	0	1
	TB	0	0	0	0	0
					Total	1

**Table 4:** Values represent the number of selected isolates identified at each treatment and fermentation time and do not represent the relative abundance of each species in the total culturable bacterial population.

Bacterial Species	Group	Treatment	0 h	48 h	96 h	144 h	Total Isolates
*Fructobacillus pseudoficulneus*	LAB *	CT	2	1	0	0	3
		TA	2	1	0	0	3
		TB	1	0	0	0	1
						Total	7
*Lentilactobacillus farraginis*	LAB	CT	1	1	0	0	2
		TA	0	0	0	0	0
		TB	0	0	0	0	0
						Total	2
*Acetobacter oryzoeni* ^1^	AAB **	CT	0	0	1	0	1
		TA	0	0	1	1	2
		TB	0	0	1	2	3
						Total	6
*Acetobacter tropicalis* ^1^	AAB	CT	0	0	0	0	0
		TA	0	0	1	1	2
		TB	0	0	0	1	1
						Total	3
*Acetobacter oryzifermentans* ^1^	AAB	CT	0	0	0	0	0
		TA	0	0	0	0	0
		TB	0	0	1	1	2
						Total	2
*Gluconobacter* sp.	AAB	CT	0	0	1	0	1
		TA	0	0	0	0	0
		TB	0	0	0	0	0
						Total	1
*Acetobacter* sp.	AAB	CT	0	0	1	1	2
		TA	0	0	2	2	4
		TB	0	0	2	2	4
						Total	10

*: Lactic acid bacteria; **: Acetic acid bacteria. ^1^: Taxonomic assignments within *Acetobacter* are reported according to the closest reference taxa based on partial 16S rRNA gene sequence similarity and phylogenetic placement. These assignments should be interpreted as *Acetobacter* spp. closely related to the indicated reference species rather than as definitive species-level identifications.

**Table 5:** Organic acid concentrations * during the fermentation treatments: CT—100% beans with pulp; TA—75% beans with pulp + 25% depulped beans; TB—50% beans with pulp + 50% depulped beans.

Compounds/ Treatments	0 h	48 h	96 h	144 h
Citric Acid (mg/g)				
CT	0.56 ± 0.11 ^a^	0.79 ± 0.17 ^a^	0.49 ± 0.14 ^a^	0.38 ± 0.02 ^b^
TA	0.47 ± 0.01 ^ab^	0.41 ± 0.01 ^b^	0.53 ± 0.01 ^a^	0.87 ± 0.23 ^a^
TB	0.30 ±0.11 ^b^	0.62 ± 0.21 ^ab^	0.55 ± 0.09 ^a^	0.73 ± 0.05 ^a^
Lactic Acid (mg/g)				
CT	0.37 ± 0.08 ^a^	0.36 ± 0.01 ^b^	0.24 ± 0.01 ^b^	0.15 ± 0.01 ^b^
TA	0.45 ± 0.01 ^a^	0.45 ± 0.02 ^a^	0.37 ± 0.08 ^a^	0.30 ± 0.03 ^a^
TB	0.27 ± 0.05 ^c^	0.25 ± 0.06 ^c^	0.33 ± 0.06 ^a^	0.20 ± 0.02 ^b^
Acetic Acid (mg/g)				
CT	0.27 ± 0.06 ^a^	0.48 ± 0.04 ^b^	1.96 ± 0.36 ^a^	1.22 ± 0.17 ^c^
TA	0.33 ± 0.10 ^a^	1.97 ± 0.17 ^a^	1.88 ± 0.28 ^a^	2.25 ± 0.02 ^b^
TB	0.29 ± 0.03 ^a^	0.40 ± 0.02 ^b^	1.37 ± 0.33 ^a^	3.30 ± 0.60 ^a^

* Values are expressed as mean ± standard deviation (*n* = 3 independent fermentation boxes). Different lowercase letters within the same fermentation time and variable indicate significant differences among treatments according to pairwise comparisons with Holm–Bonferroni adjustment (*p* < 0.05). Results in mg/g, wet basis (w.b.).

**Table 6:** Changes in sugar concentrations * during the fermentation treatments: CT—100% beans with pulp; TA—75% beans with pulp + 25% depulped beans; TB—50% beans with pulp + 50% depulped beans.

Compounds/ Treatments	0 h	48 h	96 h	144 h
Glucose (mg/g)				
CT	1.29 ± 0.06 ^a^	2.20 ± 0.13 ^a^	1.33 ± 0.06 ^a^	0.68 ± 0.01 ^a^
TA	1.01 ± 0.01 ^b^	0.44 ± 0.01 ^b^	0.47 ± 0.04 ^b^	0.61 ± 0.08 ^a^
TB	0.59 ± 0.09 ^c^	0.39 ± 0.01 ^c^	0.39 ± 0.08 ^b^	0.38 ± 0.03 ^b^
Fructose (mg/g)				
CT	2.53 ± 0.08 ^a^	3.18 ± 0.10 ^a^	2.08 ± 0.13 ^a^	1.62 ± 0.01 ^b^
TA	2.26 ± 0.01 ^b^	1.25 ± 0.03 ^b^	1.44 ± 0.08 ^b^	2.04 ± 0.04 ^a^
TB	0.92 ± 0.02 ^c^	1.03 ± 0.03 ^c^	0.73 ± 0.09 ^c^	1.03 ± 0.05 ^c^
Sucrose (mg/g)				
CT	1.21 ± 0.01 ^a^	1.39 ± 0.03 ^a^	1.31 ± 0.03 ^a^	1.25 ± 0.08 ^b^
TA	1.24 ± 0.02 ^a^	1.26 ± 0.03 ^b^	1.36 ± 0.09 ^a^	1.40 ± 0.02 ^a^
TB	1.30 ± 0.07 ^a^	1.28 ± 0.04 ^ab^	1.31 ± 0.06 ^a^	1.26 ± 0.01 ^b^

* Values are expressed as mean ± standard deviation (*n* = 3 independent fermentation boxes). Different lowercase letters within the same fermentation time and variable indicate significant differences among treatments according to pairwise comparisons with Holm–Bonferroni adjustment (*p* < 0.05). Results in mg/g, wet basis (w.b.).

## Data Availability

The original contributions presented in the study are included in the article; further inquiries can be directed to the corresponding author.

## Supplementary Materials

The following supporting information can be downloaded at: https://www.mdpi.com/article/10.3390/foods15172976/s1, Table S1. Species identification of isolated yeasts from cocoa fermentation treatments: CT—100% beans with pulp; TA—75% beans with pulp + 25% depulped beans; TB—50% beans with pulp + 50% depulped beans, in the Brazilian Amazon using ITS sequences; Table S2. Species identification of isolated bacteria from cocoa fermentation treatments: CT—100% beans with pulp; TA—75% beans with pulp + 25% depulped beans; TB—50% beans with pulp + 50% depulped beans, in the Brazilian Amazon using 16S sequence; Figure S1. Maximum parsimony phylogenetic tree based on the ITS region of yeasts of the genus Hanseniaspora. The tree was inferred using the Maximum Parsimony (MP) method, with Subtree-Pruning-Regrafting (SPR) used as the heuristic search algorithm, and 1000 bootstrap replicates. Bootstrap values are shown at the nodes. The isolate obtained in this study is highlighted in bold; Figure S2. Maximum parsimony phylogenetic tree based on the ITS region of yeasts of the genus *Pichia*. The tree was inferred using the Maximum Parsimony (MP) method, with Subtree-Pruning-Regrafting (SPR) used as the heuristic search algorithm, and 1000 bootstrap replicates. Bootstrap values above 50% are shown. Isolates sequenced in this study are highlighted in bold; Figure S3. Maximum parsimony phylogenetic tree based on the ITS region of *Saccharomyces cerevisiae* strains. The tree was inferred using the Maximum Parsimony (MP) method, with Subtree-Pruning-Regrafting (SPR) used as the heuristic search algorithm, and 1000 bootstrap replicates. Isolates sequenced in this study are highlighted in bold; Figure S4. Maximum parsimony phylogenetic tree based on the 16S rRNA gene of acetic acid bacteria of the genus *Acetobacter*. The tree was inferred using the Maximum Parsimony (MP) method, with Subtree-Pruning-Regrafting (SPR) used as the heuristic search algorithm, and 1000 bootstrap replicates. Isolates from this study are highlighted in bold; Figure S5. Maximum parsimony phylogenetic tree based on the 16S rRNA gene of lactic acid bacteria of the species *Fructobacillus pseudoficulneus*. The tree was inferred using the Maximum Parsimony (MP) method, with Subtree-Pruning-Regrafting (SPR) used as the heuristic search algorithm, and 1000 bootstrap replicates. Isolates from this study are highlighted in bold.

## Abbreviations

The following abbreviations are used in this manuscript:

AAB	Acetic acid bacteria
BLAST	Basic Local Alignment Search Tool
CT	Control fermentation treatment (beans with pulp)
HPLC	High-Performance Liquid Chromatography
LAB	Lactic acid bacteria
LOD	Limit of Detection
LOQ	Limit of Quantification
MRS	De Man, Rogosa and Sharpe
PCR	Polymerase Chain Reaction
RI	Refractive Index
SDGs	United Nations Sustainable Development Goals
TA	Fermentative treatment with 75% beans with pulp + 25% depulped beans
TB	Fermentative treatment with 50% beans with pulp + 50% depulped beans
TTA	Total Titratable Acidity
YPD	Yeast Peptone Dextrose
