# Supplementary material for: Effects of Partial Pulp Removal on Microbial Dynamics and Metabolite Profiles During On-Farm Cocoa Fermentation
Source: Foods. 2026 Aug 25;15(17):2976. doi: 10.3390/foods15172976 (PMC13565043; doi:10.3390/foods15172976)
Supplement: Supplementary file 1 [file foods-15-02976-s001.zip › foods-4466124-supplementary.pdf]

SUPPLEMENTARY MATERIAL

Effects of Partial Pulp Removal on Microbial Dynamics and Metabolite Profiles during Cocoa Fermentation

Authors: Gilson C. A. Chagas-Junior, Nelson Rosa Ferreira, Paulo Túlio de Souza Silveira, Anderson Moraes da Silva, Diego Assis das Graças, Artur Silva, Braian Saimon Frota da Silva, Dominic Wimmer, Priscilla Efraim, Alessandra Santos Lopes.

Table S1. Species identification of isolated yeasts from cocoa fermentation treatments: CT – 100% beans with pulp; TA – 75% beans with pulp + 25% depulped beans; TB – 50% beans with pulp + 50% depulped beans, in the Brazilian Amazon using ITS sequences.

GenBank Access	Yeast Species	e-value	Identification (%)
PV940350	*Saccharomyces cerevisiae*	0.0	100%
PV940351	*Saccharomyces cerevisiae*	0.0	100%
PV940352	*Saccharomyces cerevisiae*	0.0	100%
PV940353	*Saccharomyces cerevisiae*	0.0	100%
PV940354	*Saccharomyces cerevisiae*	0.0	100%
PV940355	*Saccharomyces cerevisiae*	0.0	100%
PV940356	*Pichia kudriavzevii*	0.0	100%
PV940357	*Saccharomyces cerevisiae*	0.0	100%
PV940358	*Saccharomyces cerevisiae*	0.0	100%
PV940359	*Saccharomyces cerevisiae*	0.0	100%
PV940360	*Saccharomyces cerevisiae*	0.0	100%
PV940361	*Saccharomyces cerevisiae*	0.0	100%

GenBank Access	Yeast Species	e-value	Identification (%)
PV940362	*Saccharomyces cerevisiae*	0.0	100%
PV940363	*Saccharomyces cerevisiae*	0.0	100%
PV940365	*Saccharomyces cerevisiae*	0.0	100%
PV940366	*Saccharomyces cerevisiae*	0.0	100%
PV940367	*Saccharomyces cerevisiae*	0.0	100%
PV940368	*Hanseniaspora opuntiae*	0.0	100%
PV940369	*Saccharomyces cerevisiae*	0.0	100%
PV940370	*Saccharomyces cerevisiae*	0.0	100%
PV940371	*Saccharomyces cerevisiae*	0.0	99.82
PV940372	*Saccharomyces cerevisiae*	0.0	100%
PV940373	*Saccharomyces cerevisiae*	0.0	100%
PV940374	*Saccharomyces cerevisiae*	0.0	100%
PV940375	*Saccharomyces cerevisiae*	0.0	100%
PV940376	*Saccharomyces cerevisiae*	0.0	100%
PV940377	*Saccharomyces cerevisiae*	0.0	100%
PV940378	*Saccharomyces cerevisiae*	0.0	100%
PV940379	*Saccharomyces cerevisiae*	0.0	100%

GenBank Access	Yeast Species	e-value	Identification (%)
PV940380	*Saccharomyces cerevisiae*	0.0	100%
PV940381	*Saccharomyces cerevisiae*	0.0	100%
PV940382	*Saccharomyces cerevisiae*	0.0	100%
PV940383	*Saccharomyces cerevisiae*	0.0	100%
PV940384	*Saccharomyces cerevisiae*	0.0	100%
PV940385	*Saccharomyces cerevisiae*	0.0	100%
PV940386	*Saccharomyces cerevisiae*	0.0	100%
PV940387	*Saccharomyces cerevisiae*	0.0	100%
PV940388	*Saccharomyces cerevisiae*	0.0	100%
PV940389	*Saccharomyces cerevisiae*	0.0	100%
PV940390	*Saccharomyces cerevisiae*	0.0	100%
PV940391	*Saccharomyces cerevisiae*	0.0	100%
PV940392	*Saccharomyces cerevisiae*	0.0	100%
PV940393	*Saccharomyces cerevisiae*	0.0	100%
PV940394	*Saccharomyces cerevisiae*	0.0	100%

Table S2. Species identification of isolated bacteria from cocoa fermentation treatments: CT – 100% beans with pulp; TA – 75% beans with pulp + 25% depulped beans; TB – 50% beans with pulp + 50% depulped beans, in the Brazilian Amazon using 16S sequence.

GenBank Access	Bacteria Species	e-value	Identification (%)
PX392584	*Acetobacter oryzoeni*[1]	0.0	99.64
PX392587	*Acetobacter oryzoeni*[1]	0.0	99.45
PX392589	*Acetobacter oryzoeni*[1]	0.0	99.64
PX392590	*Acetobacter oryzoeni*[1]	0.0	99.76
PX392591	*Lentilactobacillus farraginis*	0.0	98.44
PX392592	*Lentilactobacillus farraginis*	0.0	99.22
PX392593	*Acetobacter oryzoeni*[1]	0.0	99.64
PX392594	*Fructobacillus pseudoficulneus*	0.0	100
PX392595	*Fructobacillus pseudoficulneus*	0.0	99.22
PX392596	*Fructobacillus pseudoficulneus*	0.0	100
PX392597	*Fructobacillus pseudoficulneus*	0.0	100
PX392598	*Fructobacillus pseudoficulneus*	0.0	100
PX392599	*Fructobacillus pseudoficulneus*	0.0	100
PX392600	*Acetobacter tropicalis*[1]	0.0	100
PX392601	*Acetobacter sp.*	0.0	99.22
PX392602	*Acetobacter sp.*	0.0	99.77
PX392603	*Acetobacter tropicalis*[1]	0.0	100
PX392604	*Acetobacter oryzifermentans*[1]	0.0	99.23
PX392605	*Acetobacter oryzifermentans*[1]	0.0	99.42
PX392606	*Acetobacter sp.*	0.0	100
PX392607	*Acetobacter sp.*	0.0	99.81
PX392608	*Acetobacter sp.*	0.0	99.61

GenBank Access	Bacteria Species	e-value	Identification (%)
PX392609	*Acetobacter sp.*	0.0	99
PX392610	*Acetobacter tropicalis*[1]	0.0	100
PX392611	*Acetobacter sp.*	0.0	99.81
PX392613	*Acetobacter sp.*	0.0	100
PX392614	*Acetobacter oryzoeni*[1]	0.0	99.24
PX392615	*Acetobacter sp.*	0.0	99.52
PX392616	*Acetobacter sp.*	0.0	99.76
PX392617	*Gluconobacter sp.*	0.0	99.51
PX392618	*Fructobacillus pseudoficulneus*	0.0	99.74

[1]: Taxonomic assignments within *Acetobacter* are based on partial 16S rRNA gene sequence similarity and phylogenetic placement. The isolates are therefore reported as *Acetobacter* spp. closely related to the indicated reference species, rather than as definitive species-level identifications.

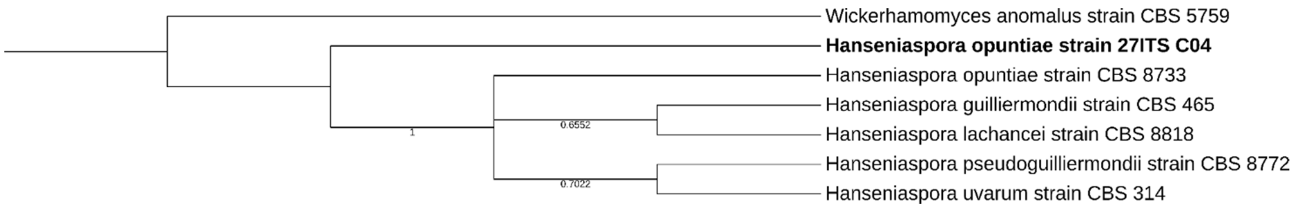

Figure S1. Maximum parsimony phylogenetic tree based on the ITS region of yeasts of the genus *Hanseniaspora*. The tree was inferred using the Maximum Parsimony (MP) method, with Subtree-Pruning-Regrafting (SPR) used as the heuristic search algorithm, and 1000 bootstrap replicates. Bootstrap values are shown at the nodes. The isolate obtained in this study is highlighted in bold.

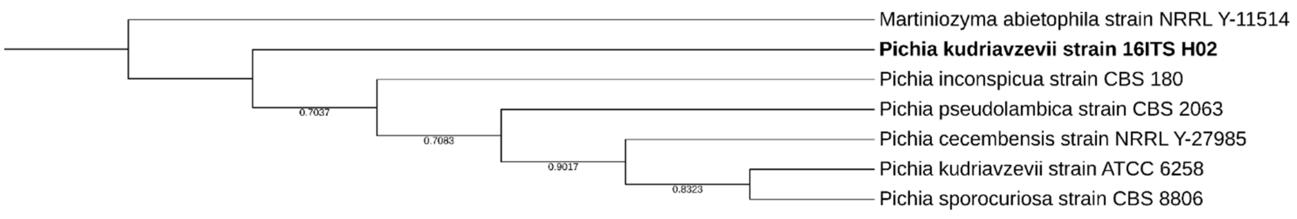

Figure S2. Maximum parsimony phylogenetic tree based on the ITS region of yeasts of the genus *Pichia*. The tree was inferred using the Maximum Parsimony (MP) method, with Subtree-Pruning-Regrafting (SPR) used as the heuristic search algorithm, and 1000 bootstrap replicates. Bootstrap values above 50% are shown. Isolates sequenced in this study are highlighted in bold.

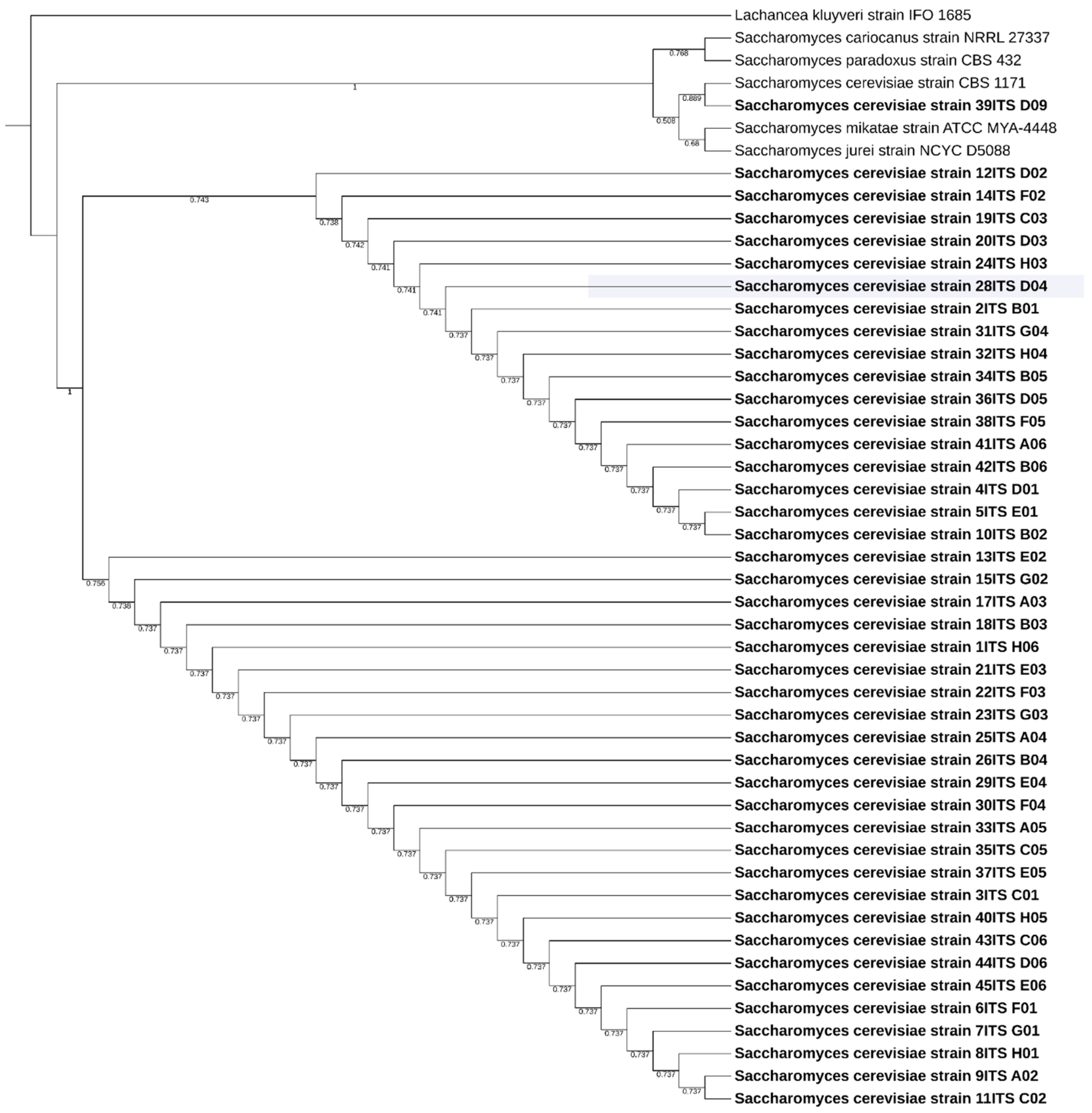

Figure S3. Maximum parsimony phylogenetic tree based on the ITS region of *Saccharomyces cerevisiae* strains. The tree was inferred using the Maximum Parsimony (MP) method, with Subtree-Pruning-Regrafting (SPR) used as the heuristic search algorithm, and 1000 bootstrap replicates. Isolates sequenced in this study are highlighted in bold.

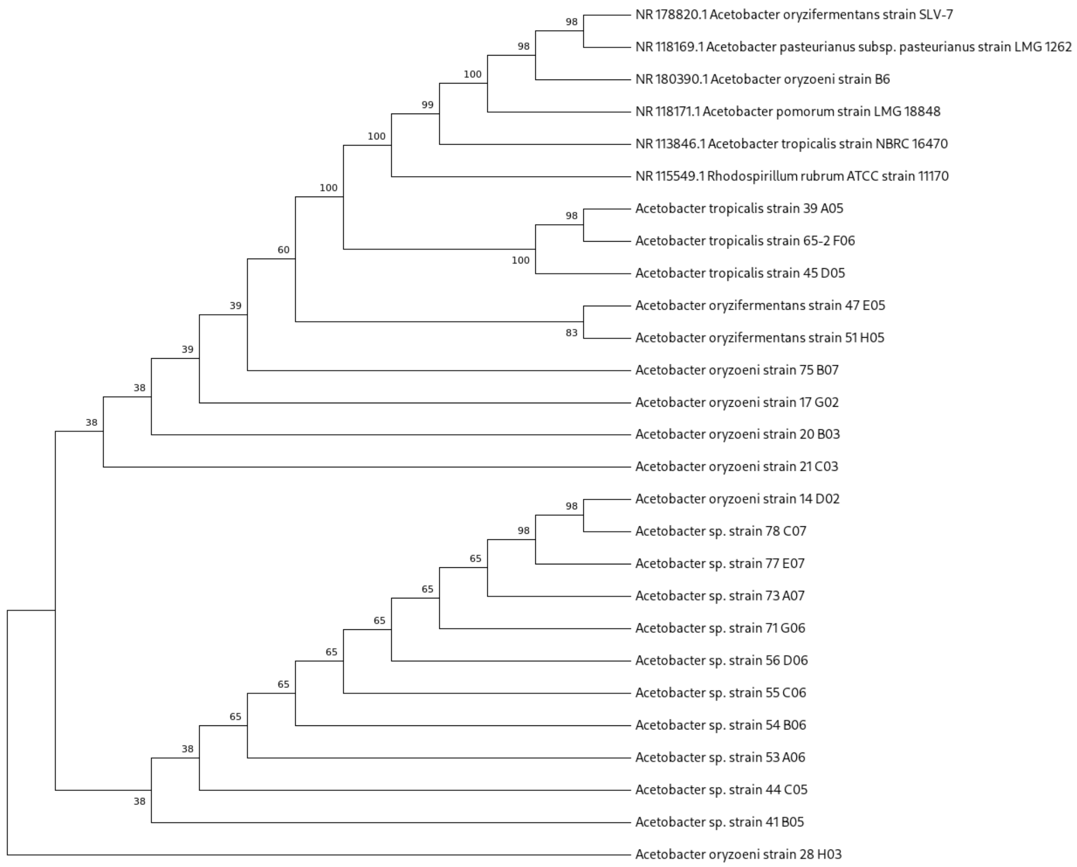

Figure S4. Maximum parsimony phylogenetic tree based on the 16S rRNA gene of acetic acid bacteria of the genus *Acetobacter*. The tree was inferred using the Maximum Parsimony (MP) method, with Subtree-Pruning-Regrafting (SPR) used as the heuristic search algorithm, and 1000 bootstrap replicates. Isolates from this study are highlighted in bold.

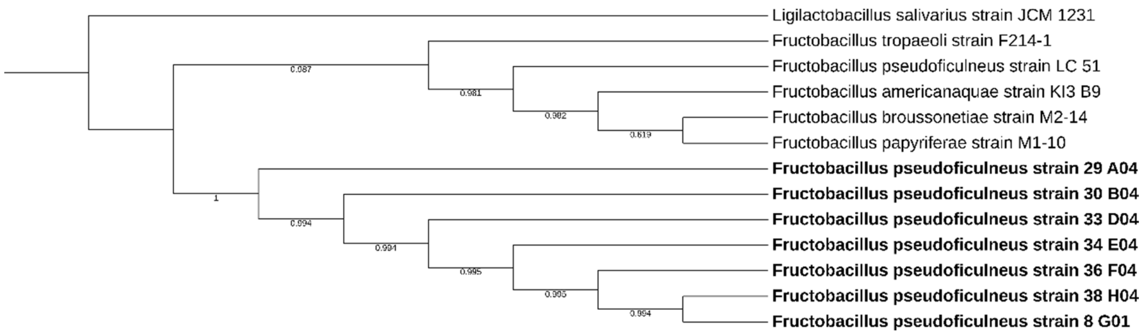

Figure S5. Maximum parsimony phylogenetic tree based on the 16S rRNA gene of lactic acid bacteria of the species *Fructobacillus pseudoficulneus*. The tree was inferred using the Maximum Parsimony (MP) method, with Subtree-Pruning-Regrafting (SPR) used as the heuristic search algorithm, and 1000 bootstrap replicates. Isolates from this study are highlighted in bold.
